# Clinical features and outcomes of 1845 patients with follicular lymphoma: a real-world multicenter experience in China

**DOI:** 10.1186/s13045-021-01139-6

**Published:** 2021-08-23

**Authors:** Jie Zha, Liyuan Fan, Shuhua Yi, Haifeng Yu, Zhong Zheng, Wei Xu, Manman Deng, Zhijuan Lin, Zhifeng Li, Lingyan Ping, Xiaohua He, Feili Chen, Ying Xie, Biyun Chen, Huilai Zhang, Li Wang, Kaiyang Ding, Wenyu Li, Haiyan Yang, Weili Zhao, Lugui Qiu, Zhiming Li, Yuqin Song, Bing Xu

**Affiliations:** 1 Department of Hematology, The First Affiliated Hospital of Xiamen University and Institute of Hematology, School of Medicine, Xiamen University, Xiamen, China; 2 Key Laboratory of Xiamen for Diagnosis and Treatment of Hematological Malignancy, Xiamen, China; 3 State Key Laboratory of Experimental Hematology, National Clinical Research Center for Blood Diseases, Blood Diseases Hospital and Institute of Hematology, Chinese Academy of Medical Sciences and Peking Union Medical College, Tianjin, China; 4 Department of Lymphoma, Cancer Hospital of the University of Chinese Academy of Sciences (Zhejiang Cancer Hospital), Hangzhou, China; 5 Department of Lymphoma, Institute of Cancer and Basic Medicine (IBMC), Chinese Academy of Sciences, Hangzhou, China; 6 Shanghai Institute of Hematology, State Key Laboratory of Medical Genomics, National Research Center for Translational Medicine at Shanghai, Ruijin Hospital Affiliated to Shanghai Jiao Tong University School of Medicine, Shanghai, China; 7 Department of Hematology, The First Affiliated Hospital of Nanjing Medical University, Jiangsu Province Hospital, Nanjing, China; 8 Key Laboratory of Carcinogenesis and Translational Research (Ministry of Education), Peking University Cancer Hospital and Institute, Beijing, China; 9 Department of Medical Oncology, Sun Yat-Sen University Cancer Center, Guangzhou, China; 10 State Key Laboratory of Oncology in South China, Guangzhou, China; 11 Collaborative Innovation Center for Cancer Medicine, Guangzhou, China; 12 Lymphoma Division, Guangdong Provincial People’s Hospital, Guangdong Academy of Medical Sciences, Guangzhou, China; 13 Shengli Clinical Medical College of Fujian Medical University, Department of Hematology, Fujian Provincial Hospital, Fujian Medical University, Fuzhou, China; 14 Department of Lymphoma, Tianjin Medical University Cancer Hospital, Tianjin, China; 15 Department of Hematology, The First Affiliated Hospital of USTC Anhui Provincial Hospital, Hefei, China

**Keywords:** Follicular lymphoma (FL), Chinese, Rituximab, Chemotherapy, Histological transformation (HT)

## Abstract

Clinical features and outcomes of FL patients in Chinese population are limited, thus promoting us to perform this analysis on a large cohort of 1845 patients with FL enrolled from nine medical centers nationwide in China. In this cohort, the median age of patients at diagnosis was 53 years, which was comparable to that reported previously for Chinese FL patients (49–51 years) but younger than that for Western FL patients (60–65 years). In contrast with Western patients, Chinese FL patients more likely involved extranodal sites but less frequently infiltrated bone marrow. Other clinical characteristics were comparable between two populations. In this study, 91% of patients were managed with chemotherapy, yielding 72% and 46% of overall-response rate and complete remission. After median 55-month follow-up, 5-year progressive-free and overall survival were 61% and 89%, respectively. Both were analogous to those reported in prior Chinese and Western studies. Consistent with published data, addition of rituximab into both induction (*R*_i_) and maintenance (*R*_m_) treatment led to the most favorable outcomes. Interestingly, *R*_i_ only had better outcomes than *R*_m_ only. Notably, 7% of patients experienced histologic transformation (HT) and correlated with poor survival. Of the transformed FL cases, 3% and 4% of HT events occurred prior to or post-treatment, respectively. Importantly, the latter displayed worse outcomes than the former. Altogether, this study provides real-world information of the largest cohort of FL patients so far in China, which might lay a foundation for clinical investigation of Chinese FL in future.

**Supplementary Information:**

The online version contains supplementary material available at 10.1186/s13045-021-01139-6.

To the editor,

Follicular lymphoma (FL), a common indolent B-cell lymphoma, is characterized by its high heterogeneity in clinical characteristics and outcomes [1]. Demographics, clinical characteristics, treatment patterns and outcomes of FL patients have been well documented in Western countries [2, 3]. However, this information is largely lacking in China. To understand clinical presentations, treatments and prognosis of Chinese FL patients, we performed a retrospective multicenter study, which enrolled 1845 patients (age > 18 years) with newly diagnosed FL between 2000 and 2020 in China. Patients and methods for this study are described in detail in Additional file 1.

Demographics and clinical characteristics of the patients enrolled in this study are summarized in Table 1. The median age at diagnosis was 53 years in our cohort, similar to that reported earlier for Chinese FL patients (49–51 years) but much younger than that reported in Western cohorts (60–65 years) [2–5]. Consistent with the prior study from China [4], Chinese FL patients had relatively lower rate of ECOG ≤ 1 than non-Chinese counterparts, reflecting poor performance status; moreover, approximately 40% of Chinese FL patients had extranodal involvement sites of > 1, significantly higher than that demonstrated in the cohorts of Western countries (5–25%) [2, 3]. Although the incidence of bone-marrow infiltration (BMI) in this cohort (28%) was higher than that reported previously for Chinese FL patients (15.2%) [4], the BMI rates in Chinese FL patients were lower than that in Western patients (29–52%) [2–4]. Other clinical features of FL patients in our cohort were comparable to those reported for Chinese FL patients and in the Western cohorts [2–5].

**Table 1:** Baseline patient and disease characteristics in the entire cohort (*n* = 1845)

Characteristics	*N* (%)
Age (median, range)	53 (18–95)
*Gender*	
Female	976 (53%)
Male	869 (47%)
*ECOG*	
0–1	1569 (85%)
2–4	105 (6%)
*Histological grade*	
1–2	1093 (59%)
3	644 (35%)
*Disease stage*	
I/II	364 (20%)
III/IV	1365(74%)
*B symptoms*	
No	792 (43%)
Yes	237 (13%)
*Lymph node* > *4*	
No	721 (39%)
Yes	996 (54%)
> *1 EN site*	
No	733 (40%)
Yes	779 (42%)
*Bulky disease*	
No	1247 (68%)
Yes	385 (21%)
*Marrow involved*	
No	1263 (68%)
Yes	521 (28%)
*HGB* < *120 g/l*	
No	1343 (73%)
Yes	423 (23%)
*LDH* > *1 ULN*	
No	1251 (68%)
Yes	481 (26%)
$\beta$*2-MG* > *3*	
No	977 (53%)
Yes	868 (47%)

Treatment patterns and therapeutic responses are detailed in Additional file 2. In our cohort, 91% of FL patients received systemic chemotherapies, among which CHOP (cyclophosphamide, doxorubicin, vincristine and prednisone) ± rituximab (R) was the most frequently used regimen, probably representing the major regimen for frontline treatment of FL in China, in accordance with the earlier report [4]. However, only 1% of patients received bendamustine plus rituximab, due to unavailability of bendamustine in China until 2019 [6]. Unlike approximately 20% of FL patients treated with observation in the cohorts of Western countries [7], only 7% of Chinese FL patients were administered with watchful waiting. In the study, overall response rate (ORR) was 72% with 46% complete remission (CR). 5-year progressive-free (PFS) and overall survival (OS) for all patients were 61% and 89%, respectively (Fig. 1A, B). Both were analogous to those observed previously in Chinese FL patients and several Western cohorts [4, 8, 9]. We found that rituximab-based induction therapy (*R*_i_) followed by rituximab maintenance (*R*_m_) resulted in the best outcomes (both PFS and OS), consistent with the findings from another real-world study in Chinese population [10]. *R*_i_ only was superior to Rm only (Fig. 1C, D). This observation suggests that incorporating rituximab into induction treatment might be more beneficial than using rituximab for maintenance if patients could not afford long-term usage of this costly agent.

**Fig. 1:**
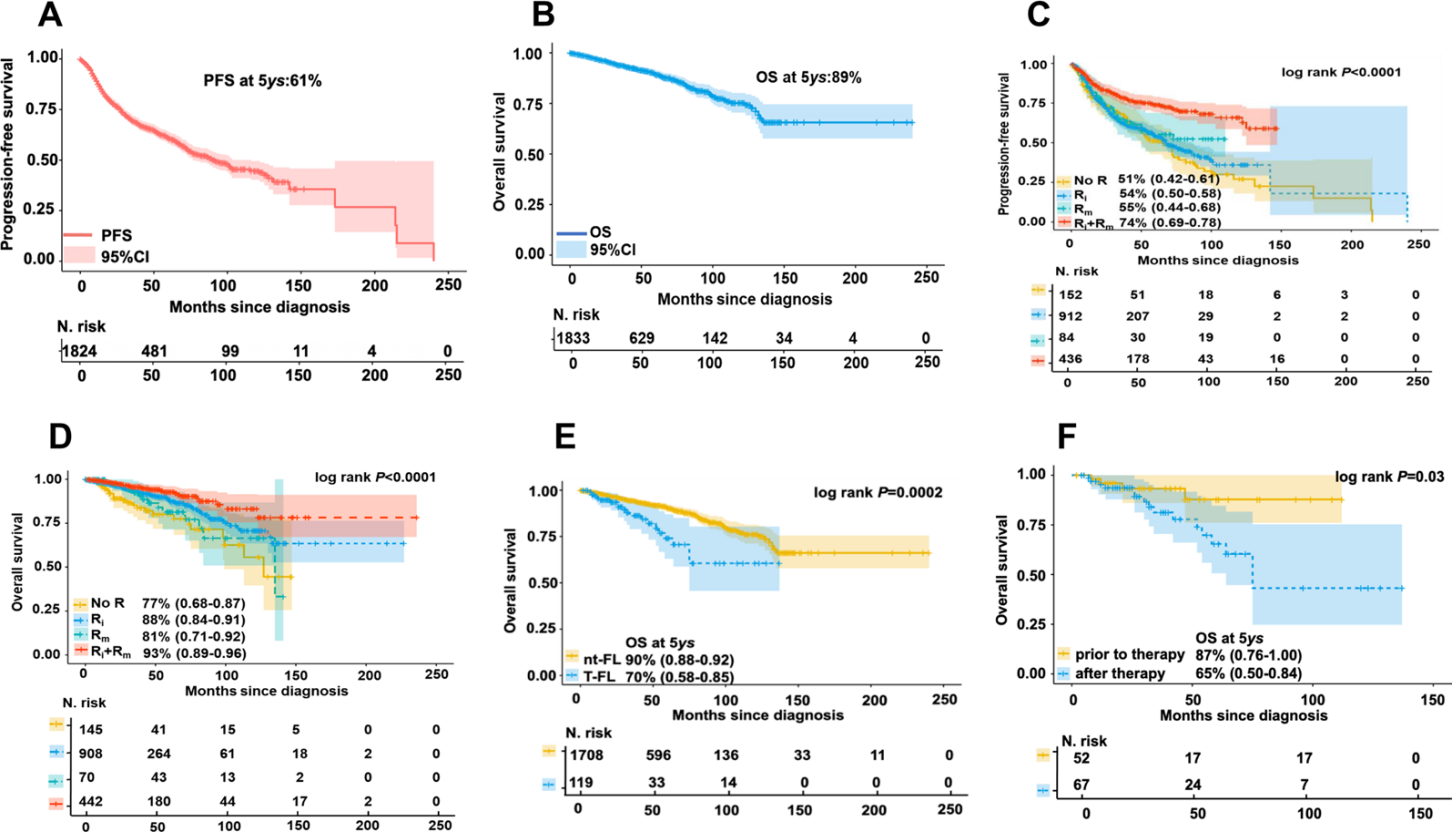
Clinical prognostic analysis of Chinese FL patients in the entire cohort and different subgroups. **A**, **B** 5-year progression-free survival (PFS, **A**) and overall survival (OS, **B**) of Chinese FL population in the whole cohort. **C**, **D** Kaplan–Meier curves of PFS (**C**) and OS (**D**) according to different rituximab administrations (no rituximab treatments (No *R*), first-line induction chemotherapies with rituximab (*R*_i_), maintenance with rituximab (*R*_m_), and *R*_i_ plus *R*_m_ regimens). **E** OS for patients with or without histological transformation (HT). **F** OS for patients with HT prior to or post-treatment

Histological transformation (HT) represents a crucial feature of FL and correlates with unfavorable outcomes [11]. Transformed FL (t-FL) can happen prior to or post-chemotherapy [12]. In our cohort, 125 patients (7%) experienced HT, of which 3% and 4% of transformed cases were observed prior to or post-treatment, respectively. Analogous to those described in previous studies involving Western countries, patients with t-FL displayed poorer outcomes than those with non-transformed FL (nt-FL; Fig. 1E; *P* = 0.0002). In this study, patients with t-FL prior to treatment had 87% of 5-year OS, which was similar to those with nt-FL but significantly better than those whose disease was transformed post-chemotherapy (Fig. 1F; *P* = 0.040). This observation suggests that patients with t-FL prior to therapy could be treated as those with nt-FL, but intensive chemotherapies should be administrated for patients with t-FL post-therapy.

This study reveals that Chinese FL patients were much younger and had higher extranodal involvement but lower BM infiltration than Western FL patients. Most Chinese FL patients received systemic chemotherapies, with CHOP ± R representing the most common regimen. In terms of ORR, CR, PFS and OS of FL patients, no significant difference was observed between our cohort and the cohorts previously reported in China and Western countries. *R*_i_ plus *R*_m_ yielded the most favorable outcome, while *R*_i_ only was superior to Rm only when they were applied separately. 7% of FL patients underwent HT, of which 3% and 4% of cases transformed prior to or post-chemotherapy. The latter had poorer outcome than the former. Collectively, this large retrospective study outlines the clinical features and outcomes of Chinese FL patients, which might lay a foundation for future clinical investigation of FL in China.

## Supplementary Information

**Additional file 1.** Patients and methods.

**Additional file 2: Table S1.** Treatment pattern and clinical response overview based on distinct therapeutic approaches.

## Data Availability

All datasets supporting the conclusions of this study are included in the figures, tables and additional files.

## Abbreviations

FL: Follicular lymphoma
ORR: Overall response rate
CR: Complete remission
HT: Histologic transformation
PFS: Progressive-free survival
OS: Overall survival
t-FL: Transformed follicular lymphoma
CHOP: Cyclophosphamide, doxorubicin, vincristine and prednisone
BR: Bendamustine plus rituximab
*R*_i_: Rituximab-based induction therapy
*R*_m_: Rituximab maintenance
nt-FL: Non-transformed FL
