# Supplementary material for: Clinical features and outcomes of 1845 patients with follicular lymphoma: a real-world multicenter experience in China
Source: J Hematol Oncol. 2021 Aug 23;14:131. doi: 10.1186/s13045-021-01139-6 (PMC8383436; doi:10.1186/s13045-021-01139-6)
Supplement: Supplementary file 1 — Additional file 1. Patients and methods. [file 13045_2021_1139_MOESM1_ESM.docx]

**Patients and Methods**

**Patients**

We performed a multicenter retrospective study of patients with newly diagnosed FL from 2000 to 2020 at 9 Chinese medical centers. This study was approved by the institutional review board of all collaborative institutions. A total of 2046 patients with FL were initially screened in the study, while 201 patients were subsequently excluded due to ineligible to our enrollment criteria, including those aged < 18 years old, diagnosed with FL3b, lacked adequate clinical information, or lost follow-up.

Diagnosis was made by the institutional hematopathology expert review board at each site, while central pathologic review was not performed. Transformation from FL to other forms of lymphomas was confirmed by biopsy. According to the institutional standard of care for FL patients, disease staging, treatment selection, and response assessment were carried out at the discretion of treating physicians.

**Variables and endpoints**

The demographic and clinical characteristics of FL patients were assessed at initial diagnosis, including age, sex, performance status (PS), disease stage, histological grade, B symptoms, involved lymph nodes (LNs), extranodal disease, bone marrow involvement, bulky disease; laboratory examination included serum lactate dehydrogenase (LDH), hemoglobin (HGB), and $\beta$2-microglobulin (β2-MG). Treatment response was classified as complete remission (CR), partial response (PR), stable disease (SD), or progression of disease (PD), according to the Lugano criteria [1]. Overall survival (OS) was defined as the time from diagnosis to death or last follow-up. Progressive-free survival (PFS) was defined as the time from diagnosis to progression, relapse, death from any cause, or last follow-up.

**Statistical analysis**

Clinicopathologic characteristics between patients were compared using Wilcoxon rank-sum tests for continuous variables and $\chi$2 tests for categorical variables. Median follow-up was determined by reverse Kaplan-Meier analysis. Kaplan-Meier curve was used to estimate PFS and OS. Proportional hazards regression was used to estimate the effect of risk factors on PFS and OS, for which the results are presented as hazard ratios (HRs) together with 95% confidence intervals (CIs). *P* values for these analyses were calculated by Wald tests.

Reference

1. Cheson BD, Fisher RI, Barrington SF, Cavalli F, Schwartz LH, Zucca E, et al. Recommendations for initial evaluation, staging, and response assessment of Hodgkin and non-Hodgkin lymphoma: the Lugano classification. *J Clin Oncol* 2014;**32**(27):3059-68.
