# Supplementary material for: Clinical features and outcomes of 1845 patients with follicular lymphoma: a real-world multicenter experience in China
Source: J Hematol Oncol. 2021 Aug 23;14:131. doi: 10.1186/s13045-021-01139-6 (PMC8383436; doi:10.1186/s13045-021-01139-6)
Supplement: Supplementary file 2 — Additional file 2: Table S1. Treatment pattern and clinical response overview based on distinct therapeutic approaches. [file 13045_2021_1139_MOESM2_ESM.docx]

**Supplementary Table S1. Treatment pattern outline and clinical responses based on distinct treatment regimens**

| **Treatment** | **Patients, n** | **ORR %** | **CR %** | **CR, n** | **PR, n** | **SD, n** | **PD, n** |
| --- | --- | --- | --- | --- | --- | --- | --- |
| CHOP±R | 1450 | 79 | 51 | 744 | 402 | 75 | 76 |
| CVP±R | 27 | 67 | 28 | 8 | 10 | 4 | 2 |
| R2 | 45 | 62 | 29 | 13 | 15 | 8 | 5 |
| BR | 23 | 65 | 35 | 8 | 7 | 4 | 2 |
| R | 142 | 65 | 39 | 56 | 35 | 8 | 7 |
| Radiotherapy alone | 36 | 75 | 72 | 26 | 1 | 2 | 0 |
| No therapy | 122 | - | - | - | - | - | - |
| Total | 1845 | 72 | 46 | 855 | 470 | 101 | 92 |

ORR objective response rate, CR complete response, PR partial response, SD stable disease, PD progressive disease
